# Physical Activity Is Associated with Physical Fitness and Executive Function among School Children in the Jiangxi Region of China

**DOI:** 10.3390/children11010042

**Published:** 2023-12-29

**Authors:** Renle Wu, Sunga Kong, Suh-Jung Kang

**Affiliations:** 1 College of Physical Education, Yichun University, Yichun 336000, China; 202255@jxycu.edu.cn; 2 Samsung Advanced Institute of Health Sciences & Technology (SAIHST), Sungkyunkwan University, Seoul 06355, Republic of Korea; 3 Sports and Health Care Major, College of Culture and Arts, Sangmyung University, Seoul 03016, Republic of Korea

**Keywords:** children, executive function, physical activity, physical fitness, school

## Abstract

Previous studies have suggested that a positive relationship exists between physical activity (PA), physical fitness, and cognitive function in children and adolescents. However, research remains limited on the relationship among these three variables in Chinese individuals not living in big cities or specific regions. Therefore, this study investigated the association of PA with physical fitness and cognitive function (specifically, executive function) among 1100 children aged 9–12 years living in the Jiangxi region of China. Participants’ PA levels were measured using the PA questionnaire for older children. Physical fitness was assessed based on cardiorespiratory endurance, muscle strength, muscular endurance, flexibility, and body mass index (BMI). Executive function was assessed using the Behavior Rating in Inventory of Executive Function questionnaire. Data were analyzed using one-way analyses of variance, Scheffé tests, one-way analyses of covariance, and Pearson’s correlation coefficients. The results showed that PA is significantly associated (*p* < 0.01) with cardiorespiratory endurance (r = 0.460), muscular endurance (r = 0.270), muscle strength (r = 0.190), BMI (r = −0.114), and executive function (r = −0.140). Moreover, all components of physical fitness and executive function differed significantly based on PA level, with and without age and sex as covariates (*p* < 0.05). Overall, our results highlight the importance of higher PA levels during childhood to improve physical fitness and cognitive function. Including the goal of improving health-related fitness factors, such as cardiorespiratory endurance and muscle strength, is particularly important in PA programs for children.

## 1. Introduction

The number of health problems caused by physical inactivity continues to increase worldwide. A recent study has shown that physical inactivity during the 2019 coronavirus pandemic has had a negative impact on mental and physical health, leading to anxiety, depression, stress, insomnia, fatigue, and mental health problems [1]. These problems can extend into adulthood [2]. Studies have shown that physical activity (PA) in childhood and adolescence has a positive effect on physical fitness [3], psychological well-being [4], and academic performance [5] and helps to prevent overweight and obesity [6,7]. PA is also associated with the prevention of chronic diseases in adulthood [8]. Studies have also shown that the longer one participates in PA during childhood and adolescence, the more one participates in PA in adulthood [9]. Therefore, participation in PA during childhood can have a strong impact on health throughout life.

In 2020, the World Health Organization recommended that children and adolescents should engage in at least 60 min of moderate-to-high-intensity PA and less than 2 h of screen time daily [10]. However, less than 22% of Chinese children exercise in such a manner [11], and 35% have more than 2 h of screen time daily [12,13]. The rate of physical inactivity continues to increase [14], revealing an overall decline in the PA of school-aged children in China. According to a recent study, the rate of overweight and obesity among children and adolescents in China is 20.5% [15]. This rate increased from 0.1% in 1985 to 7.3% in 2014 [16]. Notably, the likelihood of becoming obese in adulthood increases by five times if one was obese during childhood and adolescence. As obesity in childhood and adolescence persists into adulthood [17], its management is urgent. Accordingly, the Chinese government has aimed to reduce the overweight and obesity incidence among children to 28% by 2030 [18] by enacting the “Healthy China 2030” initiative, the first PA and health consensus statement for children and adolescents in China, which aims to boost the PA levels of children and adolescents and improve their physical fitness [19].

Previous studies have highlighted decreased PA and physical fitness in Chinese children and adolescents, even before the 2019 coronavirus disease pandemic [19]. Physical fitness in adolescence is considered an important indicator of health [20]. However, awareness of physical fitness and its management remain limited. A systematic review examining the association between PA, physical fitness, and body mass index (BMI) found causal relationships between PA and obesity, between physical fitness and obesity, and among obesity, PA, and physical fitness. It also emphasized the need for additional research to clarify these associations [21]. However, basic data on the physical fitness of children and adolescents in China are lacking. In addition, studies on the association of PA with physical fitness and the differences in physical fitness according to PA level are needed.

Children with a high PA level and aerobic capacity reportedly have better brain structure and function, with larger brain volumes in the basal ganglia and hippocampus, which are related to cognitive control and memory [22]. PA is related to executive function, information processing speed, language, and memory, and has also been found to be positively correlated with hippocampal capacity and memory in children [23]. PA and changes in white matter networks lead to physiological adaptations such as increased blood volume and enhanced fat mobilization ability [22,23]. In addition, a systematic review highlights the significant correlation among PA, physical fitness, and cognitive function, emphasizing their relationship in children [24]. Higher aerobic fitness is associated with superior cognitive processing speed [25], and moderate-to-vigorous PA contributes to improved executive function [26,27]. Although the direct effects of PA on various changes such as in PA, physical fitness, and cognitive function remain inconclusive, there seems to be an indirect positive impact. However, its influence on academic performance remains controversial, requiring further research [24,28,29].

Overall, existing research mainly focuses on the association with fitness in China, leaving limited exploration of cognitive function. This study aims to analyze the association of PA with physical fitness, and cognitive function among children in the Jiangxi region of China, beyond urban areas. Since childhood is an important time to learn and form a healthy lifestyle, the results of this study can be used by educators, researchers, and healthcare experts at schools as basic data for health promotion guidance, education, policy making, and research to improve PA. We hypothesized that higher PA would be associated with better physical fitness and cognitive function.

## 2. Materials and Methods

### 2.1. Participants and Data Collection

The participants in this study were children aged 9–12 years from five elementary schools in Jiangxi, China. At each school, we randomly selected two grades from the 4th, 5th, and 6th grades to select the participants from. Among the students who wished to participate in the study, 1200 students were recruited. We conducted a survey and test to determine their PA, executive function, and physical fitness levels. We excluded 68 students because they did not complete the survey or were judged to be fit and 32 students because they did not complete the physical fitness tests. As a result, we used the data from 1100 students in the analysis. This procedure was conducted after obtaining consent from the parents of the participants and approval from the Institutional Review Board of Sun Kyun Kwan University (SKKU 2023-09-011).

### 2.2. Measurements

We assessed participants’ physical characteristics, PA level, physical fitness, and executive function and recruited instructors who could provide guidance. We provided training to the instructors to increase the level of internal reliability between measurers.

#### 2.2.1. Physical Characteristics

We measured the height (cm) and weight (kg) of the participants (MG001; Meilen, China) and calculated their BMI (kg/m^2^).

#### 2.2.2. Physical Activity Level

We used the Chinese version of the PA Questionnaire for Older Children (PAQ-C) to determine participants’ PA levels [30]. This questionnaire has been found to be reliable and valid [30]. To obtain more accurate responses, the participants were asked to complete the questionnaire with one of their parents. Among the items in the PAQ-C, we used 9 of 10 items to assess physical activities performed during physical education classes, recess, lunchtime, after school, and in the evenings and weekends. We assigned a score corresponding to the answer to each item and summed all scores to calculate the total score. After calculating the total score, we classified participants based on their total score. Participants with a total score of 2 points or less (total score ≤ 2) were classified as the low PA group (*n* = 150). Those with a total score of more than 2 but equal to or less than 3 (2 < total score ≤ 3) were classified as the moderate PA group (*n* = 658). Participants with a total score more than 3 (total score > 3) were classified as the high PA group (*n* = 292) [31].

#### 2.2.3. Physical Fitness

Physical fitness was evaluated based on the fitness guidelines provided by the Korea Ministry of Culture, Sports, and Tourism for children [32].

##### Cardiorespiratory Endurance

We assessed participants’ cardiorespiratory endurance using a 15 m shuttle run test. Using the sound source used for this test, we installed two cones at a distance of 15 m apart. After hearing the sound signal at one cone, the participant ran to the other cone and returned to the starting point after hearing the sound signal again. We ensured that the participant did not start before the sound signal and recorded the total number of round trips (laps).

##### Muscle Strength

We assessed participants’ muscle strength based on their relative grip strength using a grip strength meter (TKK-4601a; Takei, Japan). The examiner measured participants’ right and left sides twice and recorded the highest value to the nearest 0.1 kg. Then, relative grip strength was evaluated using the following formula: grip strength(kg)/body mass(kg) × 100.

##### Muscle Endurance

We measured participants’ muscle endurance based on curl-ups. From a lying position with bent knees, the participants were asked to raise their upper body with outstretched arms until the fingers of both hands touched the knees. This movement was repeated in accordance with a beep that sounded once every 3 s, and we recorded the maximum number of repetitions that could be performed.

##### Flexibility

We assessed participants’ flexibility based on the sit and reach test. While sitting on the manual left forward flexion measurement device, participants were asked to bend their upper body without bending their knees, so that both hands could be placed on the scale as much as possible. At this time, we measured the value (cm) at which participants were able to stay for at least 2 s and adopted the highest value after two attempts.

#### 2.2.4. Cognitive Function

We evaluated participants’ cognitive function based on their executive function, which was measured using the Behavior Rating Inventory of Executive Function [33]. This questionnaire asks parents of children and adolescents aged 6–18 years to answer questions about their child’s behavior over the past 6 months. It comprises 86 questions: 14 questions related to individual clinical practice, 28 questions related to the behavior regulation index (BRI), and 44 questions related to the metacognition index (MI). The researcher wrote a guide and delivered it along with the questionnaire to the parents of the participants so that they could understand and complete the questionnaire more effectively. We calculated BRI and MI scores for all questions, and the two values were added to calculate the Global Executive Composite (GEC) score. This GEC score was used to determine participants’ executive function levels. All raw scores were converted to T-scores and used in data analysis [34]. Lower final scores denoted better executive function. The reliability of the Chinese version of the questionnaire lies within an acceptable range [35].

### 2.3. Data Analysis

SPSS version 22.0 (IBM Corp., Armonk, NY, USA) was used for data analysis. We performed one-way analyses of variance and Scheffé tests to compare physical fitness and cognitive function in the three PA groups. We also made this comparison excluding the influence of sex and age by performing one-way analyses of covariance and pairwise comparisons, setting sex and age as covariates. Furthermore, Pearson’s correlation coefficients were used to determine the relationship among PA, components of physical fitness, and executive function. Statistical significance was set at *p* < 0.05.

## 3. Results

### 3.1. Participant Characteristics

Table 1 presents the general characteristics of the participants. The groups differed significantly based on sex, age, and PAQ-C score (*p* < 0.05).

### 3.2. Correlation between Physical Activity, Physical Fitness, and Executive Function

Table 2 presents the results of analyzing the correlations among PA, BMI, physical fitness components, and executive function. PA was positively correlated with cardiorespiratory endurance (r = 0.460, *p* < 0.001), muscle strength (r = 0.190, *p* < 0.001), muscle endurance (r = 0.270, *p* < 0.001), BMI (r = −0.114, *p* < 0.001), and executive function (r = −0.140, *p* < 0.001).

### 3.3. Differences in Physical Fitness Based on Physical Activity Level

Table 3 shows the results of comparing physical fitness based on PA levels. All components of physical fitness differed significantly based on PA levels (*p* < 0.05). Table 4 shows the results of comparing physical fitness based on PA levels with sex and age as covariates. All components of physical fitness differed significantly based on PA levels (*p* < 0.05).

### 3.4. Differences in Executive Function Based on Physical Activity Level

Table 5 presents the results of comparing executive function based on PA levels. Table 6 presents the results of comparing executive function based on PA levels with sex and age as covariates. As previously stated, the BRI and MI scores were used to calculate the GEC score. The GEC score differed significantly based on PA levels regardless of whether sex and age were set as covariates (*p* < 0.05).

## 4. Discussion

We aimed to examine the relationship among PA levels, physical fitness, and cognitive function among children living in the Jiangxi region of China. The results showed that physical fitness and cognitive function differ depending on the level of PA. Moreover, physical fitness and cognitive function improve as the level of PA increases from low to moderate to high.

Physical fitness is a strong indicator of health in childhood and adolescence [20]. Among its components, body composition can affect physical fitness in childhood [36,37,38] and motor condition [39] and is known to be related to the level of PA [40,41]. Our study found similar results in that BMI decreased as the level of PA increased, and there was an inverse relationship between PA and BMI. Higher levels of PA appeared to be related to physical fitness components, emphasizing the need to promote PA during childhood. Furthermore, in previous studies, even when BMI was higher or lower than the normal range [42] and in the case of preschool-aged children [43], higher PA levels showed an association with lower BMIs. Furthermore, low PA levels result in weight gain and obesity, which also inhibit movements and lead to inactivity, negatively impacting physical fitness. Accordingly, the results of this study indicate that it is important to engage in appropriate levels of PA and maintain BMI within the normal range during one’s growth period.

Among the components of physical fitness, cardiorespiratory endurance provides various health benefits to adults [44], children, and adolescents [20] and reflects children’s PA habits and disease status [45]. Cardiorespiratory endurance during childhood can provide insights into the current and future health status [46], and low cardiorespiratory endurance during childhood can increase the risk of cardiovascular metabolic diseases in adulthood [47]. In this study, cardiorespiratory endurance differed depending on the level of PA; it improved with an increase in the level of PA. The results also showed that PA is positively related to cardiorespiratory endurance. Previous studies have found that one can obtain metabolic benefits if one’s muscular strength is excellent not only during adulthood but also during childhood [48], and greater muscular strength leads to a better metabolic levels [49]. Meanwhile, some studies have argued that metabolic risk has a stronger relationship with muscular strength than with cardiorespiratory endurance [50], highlighting the importance of muscular strength and endurance during childhood. One study followed-up with preschoolers for 12 months and analyzed the relationship between PA and muscle fitness. It found that moderate-to-vigorous and vigorous PA is associated with a higher fat-free mass index, suggesting that PA levels above the moderate level can have a long-term effect on muscular fitness [51]. As this study was a cross-sectional study, it was difficult to confirm the duration or intensity of PA. However, it found that the more PA, the better the muscular strength and endurance, as found in previous studies. This result was also found for other components of physical fitness, similar to the findings of previous studies that high levels of PA are related to health benefits. Even when we excluded the influence of sex and age, we obtained similar results. Thus, the results of this study suggest that increasing children’s PA positively affects several physical fitness components that have important implications for one’s health.

Executive function, attention, and memory are generally used to evaluate cognitive function. Among these, executive functions are thinking skills that assist with reasoning, planning, problem-solving, and managing one’s life and education [52]. Additionally, executive function is a key factor in the learning processes of children and adolescents and is positively associated with academic achievement [26,27]. This study found that executive function differs depending on the level of PA. It improves with an increase in the level of PA, indicating a significant relationship. Similar results were obtained when we excluded the influence of sex and age. These results align with the findings of previous studies that PA is significantly related to cognitive ability [53] and positively affects academic achievement [24]. They also correspond with previous findings that acute [53] and longitudinal [54,55] PA can improve cognitive function. This study also found that the more PA, the better the association between physical fitness and cognitive function. One study analyzed the dose association of PA. Its results could not clearly reveal the dose effect on PA, but they revealed that a daily average of 60 min of moderate-to-vigorous PA or more is more beneficial than 60 min of moderate-to-vigorous PA [56]. It can be seen that the results of this study are consistent with those of previous studies. As in this study, the mechanism through which PA improves children’s and adolescents’ cognitive function remains unclear in previous studies. However, some studies have explained that physical exercise increases blood circulation, which causes more oxygen to be supplied to the brain and nutrients to reach the brain [57]. PA, including sports, activates the motor, cardiovascular, respiratory, hormonal, immunological, and nervous systems. Therefore, it stimulates the maturation of the motor area of the brain, affects motor development, and increases the speed of nerve impulse transmission [58]. Additionally, it stimulates the secretion of neurohormones, which can be interpreted as exerting a significant effect on the stimulation of neurons that form synapses. As a result, this increases the speed of nerve impulse transmission and enhances executive function [57]. Although this study was limited to children living in specific regions, the results confirm the positive and beneficial effects of PA. Moreover, they can be used to improve the PA levels of Chinese children. To improve and maintain children’s health, strategies are needed that increase motivation and promote participation in programs, increase awareness, and increase children’s PA levels. The results of this study also show that high PA levels are associated with better cardiorespiratory endurance, muscular strength, muscular endurance, and executive function. Based on these results, a positive relationship may exist between physical fitness levels and executive function. Previous studies have found a positive relationship between physical fitness factors and cognitive function [59]. Among different factors of cognitive function, executive function has been found to have the strongest correlation with physical fitness and PA [24]. Furthermore, meta-analyses and review studies have reported that longitudinal PA is related to more factors of cognitive function than acute physical programs in the case of pre-adolescent children [60]. These previous findings indicate that physical fitness factors improve cognitive function, and more factors of cognitive function are positively impacted when one engages in PA over a long period rather than once. These findings partially support the positive relationship between PA, physical fitness, and cognitive function found in this study and demonstrate that participating in PA can positively affect cognitive function.

This study had some limitations. First, we did not utilize any specific analyses or objective measurements of PA. Most physical activities of Chinese adults take place at work or home but not during their leisure time [61,62]. Since adults’ lifestyle habits can be applied to children and infants, a detailed analysis of children’s PA, including PA in their leisure time, is necessary to understand the specific characteristics of PA. Second, since this was a cross-sectional study, causal associations could not be revealed. Third, this study did not analyze the social, environmental, economic, and genetic determinants of executive function. However, this study holds significance because it included 1100 children, and it investigated executive function using a highly reliable and valid questionnaire. 

Finally, this study focused on the Jangxi region of China; therefore, the data might not be representative of China overall. However, this study is the first investigation on the correlation between PA, physical fitness, and cognitive function among children specifically in the Jangxi region. Despite its limitation regarding national representation, our findings have significance in that they shed light on these correlations within this specific region of China, providing crucial insights into the children in this area. Furthermore, the outcomes gleaned from this investigation may serve as fundamental groundwork for formulating regional health policies and enhancing health education and programs within local schools.

## 5. Conclusions

PA in childhood is necessary for physical fitness and cognitive function. Associating higher PA levels with a healthy lifestyle and behavior can positively impact physical fitness and cognitive function. Overall, the results of this study suggest that including the goal of improving health-related fitness factors, such as cardiorespiratory endurance, muscular strength, and muscular endurance, in PA programs for children is important.

## Figures and Tables

**Table 1:** General characteristics of the participants.

Variable	Sex	LPAG (*n* = 150)	MPAG (*n* = 658)	HPAG (*n* = 292)	*p*
Sex	Male	77	320	192	<0.001 ***
	Female	73	338	100	
Age (years)	Male	10.06 ± 0.87	10.48 ± 0.92	10.65 ± 0.89	0.002 **
	Female	10.49 ± 1.02	10.50 ± 0.94	10.51 ± 0.85	
	Total	10.27 ± 0.97	10.49 ± 0.93	10.60 ± 0.88	
Score on the PAQ-C	Male	1.72 ± 0.24	2.55 ± 0.28	3.52 ± 0.40	<0.001 ***
	Female	1.75 ± 0.21	2.53 ± 0.26	3.34 ± 0.29	
	Total	1.73 ± 0.23	2.54 ± 0.27	3.46 ± 0.37	

Data are expressed as means ± standard deviations. ** *p* < 0.01; *** *p* < 0.001: tested using one-way analysis of variance and the Scheffé test. PAQ-C: Physical Activity Questionnaire for Older Children, LPAG: low physical activity group, MPAG: moderate physical activity group, HPAG: high physical activity group.

**Table 2:** Correlation between physical activity, components of physical fitness, and executive function.

	Physical Fitness					Cognitive Function
	Body Mass Index (kg/m^2^)	Cardiorespiratory Endurance (Laps)	Muscle Strength (%)	Muscle Endurance (Repetitions)	Flexibility (cm)	Executive Function (Score)
Physical Activity (score)	−0.114 (<0.001 ***)	0.460 (<0.001 **)	0.190 (<0.001 **)	0.270 (<0.001 **)	0.050 (0.097)	−0.140 (<0.001 ***)

** *p* < 0.01, *** *p* < 0.001: tested using Pearson correlation analysis.

**Table 3:** Differences in physical fitness based on the level of physical activity level.

	LPAG	MPAG	HPAG	F	*p*
Body mass index (kg/m^2^)	18.40 ± 3.95 ^a,b^	17.44 ± 3.31	16.97 ± 2.66	9.667	<0.001 ***
Fifteen-meter shuttle run test (laps)	35.54 ± 10.04 ^a,b^	45.84 ± 12.42 ^b^	56.13 ± 12.02	155.454	<0.001 ***
Relative grip strength (%)	41.17 ± 10.72 ^a,b^	44.95 ± 9.33 ^b^	48.04 ± 9.18	26.834	<0.001 ***
Curl-up (repetitions)	20.26 ± 9.09 ^b^	25.87 ± 10.11 ^b^	31.19 ± 12.24	55.803	<0.001 ***
Sit and reach (cm)	7.70 ± 6.38 ^b^	8.67 ± 6.55	9.37 ± 6.31	3.366	0.035 *

Data are expressed as means ± standard deviations. * *p* < 0.05; *** *p* < 0.001: tested using one-way analysis of variance and the Scheffé test. ^a^ *p* < 0.05 compared with the MPAG, ^b^ *p* < 0.05 compared with the HPAG. LPAG: low physical activity group, MPAG: moderate physical activity group, HPAG: high physical activity group.

**Table 4:** Differences in physical fitness based on physical activity level with sex and age as covariates.

Variable	SS	df	MS	F	*p*	Post-Hoc
Body mass index (kg/m^2^)	67.538	1	67.538	6.426	<0.011 **	a–b *, a–c ***
Fifteen-meter shuttle run test (laps)	1140.957	1	1140.957	7.948	<0.05 **	a–b ***, a–c ***, b–c ***
Relative grip strength (%)	172.388	1	172.388	1.915	0.167	a–b ***, a–c ***, b–c ***
Curl-up (repetitions)	2126.391	1	2126.391	19.240	<0.001 ***	a–b ***, a–c***, b–c ***
Sit and reach (cm)	7422.007	1	7422.007	211.721	<0.001 ***	a–c, b–c ***

a, b, and c denote low, moderate, and high physical activity level groups, respectively. SS: sum of squares, df: degrees of freedom, MS: mean square. * *p* < 0.05; ** *p* < 0.01;*** *p* < 0.001: tested using one-way analysis of covariance and pairwise comparison.

**Table 5:** Differences in executive function based on physical activity level.

	LPAG	MPAG	HPAG	F	*p*
BRI score	50.03 ± 7.96 ^a,b^	48.33 ± 8.28 ^b^	46.89 ± 7.40	7.884	<0.001 ***
MI score	54.44 ± 7.89 ^a,b^	50.62 ± 9.14 ^b^	49.28 ± 8.84	16.901	<0.001 ***
GEC score	52.87 ± 7.27 ^a,b^	49.70 ± 8.70 ^b^	48.20 ± 8.14	15.445	<0.001 ***

Data are expressed as means ± standard deviations. *** *p* < 0.001: tested using one-way analysis of variance and the Scheffé test. ^a^ *p* < 0.05 compared with the MPAG, ^b^ *p* < 0.05 compared with the HPAG. BRI: behavior regulation index, MI: metacognition index, GEC: global executive composite, LPAG: low physical activity group, MPAG: moderate physical activity group, HPAG: high physical activity group.

**Table 6:** Differences in executive function based on physical activity level with sex and age as covariates.

Variable	SS	df	MS	F	*p*	Post-hoc
BRI score	438.176	1	438.176	6.863	0.009 **	a-b *, a-c ***
MI score	1605.944	1	1605.944	20.628	<0.001 ***	a-b *, a-c ***
GEC score	1383.084	1	1383.084	20.052	<0.001 ***	a-b *, a-c ***

* *p* < 0.05; ** *p* < 0.01, *** *p* < 0.001: test using one-way analysis of covariance and pairwise comparison. SS: sum of squares, df: degrees of freedom, MS: mean square, BRI: behavior regulation index, MI: metacognition index, GEC: global executive composite. a, b, and c denote low, moderate, and high physical activity groups, respectively.

## Data Availability

The datasets used and/or analyzed in the current study are available upon reasonable request from the corresponding author (Suh-Jung Kang).
